# Availability of Alcohol on an Online Third-Party Delivery Platform Across London Boroughs, England: Exploratory Cross-Sectional Study

**DOI:** 10.2196/54587

**Published:** 2024-06-28

**Authors:** Casey Sharpe, Saloni Bhuptani, Mike Jecks, Nick Sheron, Clive Henn, Robyn Burton

**Affiliations:** 1 Office for Health Improvement and Disparities London United Kingdom; 2 Department of Public Health, Environments and Society London School of Hygiene and Tropical Medicine London United Kingdom; 3 Islington Public Health Islington Council London United Kingdom; 4 The Roger Williams Institute of Hepatology Kings College London London United Kingdom; 5 Institute of Psychiatry, Psychology, and Neuroscience Kings College London London United Kingdom; 6 Institute for Social Marketing and Health University of Stirling Stirling United Kingdom

**Keywords:** alcohol, availability, online, third-party delivery platforms, England, cross-sectional study, exploratory, licensing, public health, policy

## Abstract

**Background:**

Higher availability of alcohol is associated with higher levels of alcohol consumption and harm. Alcohol is increasingly accessible online, with rapid delivery often offered by a third-party driver. Remote delivery and online availability are important from a public health perspective, but to date, relatively little research has explored the availability of alcohol offered by online platforms.

**Objective:**

This cross-sectional exploratory study describes the availability of alcohol on the third-party platform Deliveroo within London, England.

**Methods:**

We extracted the number of outlets offering alcohol on Deliveroo for each London borough and converted these into crude rates per 1000 population (18-64 years). Outlets were grouped as outlets exclusively selling alcohol, off-licenses, and premium. We calculated Pearson correlation coefficients to explore the association between borough’s crude rate of outlets per 1000 population and average Indices of Multiple Deprivation (IMD) 2019 scores. We extracted the number of outlets also selling tobacco or e-cigarettes and used non-Deliveroo drivers. We searched addresses of the top 20 outlets delivering to the most boroughs by outlet type (60 total) to determine their associated premise.

**Results:**

We identified 4277 total Deliveroo-based outlets offering alcohol across London, including outlets delivering in multiple boroughs. The crude rate of outlets per 1000 population aged 18-64 years was 0.73 and ranged from 0.22 to 2.29 per borough. Most outlets exclusively sold alcohol (3086/4277, 72.2%), followed by off-licenses (770/4277, 18.0%) and premium (421/4277, 9.8%). The majority of outlets exclusively selling alcohol sold tobacco or e-cigarettes (2951/3086, 95.6%) as did off-licenses to a lesser extent (588/770, 76.4%). Most outlets exclusively offering alcohol used drivers not employed by Deliveroo (2887/3086, 93.6%), and the inverse was true for premium outlets (50/421, 11.9%) and off-licenses (73/770, 9.5%). There were 1049 unique outlets, of which 396 (37.8%) were exclusively offering alcohol—these outlets tended to deliver across multiple boroughs unlike off-licenses and premium outlets. Of outlets with confirmed addresses, self-storage units were listed as the associated premise for 85% (17/20) of outlets exclusively offering alcohol, 11% (2/19) of off-licenses, and 12% (2/17) of premium outlets. We found no significant relationship between borough IMD scores and crude rate of outlets per 1000 population overall (*P*=.87) or by any outlet type: exclusively alcohol (*P*=.41), off-license (*P*=.58), and premium (*P*=.18).

**Conclusions:**

London-based Deliveroo outlets offering alcohol are common and are sometimes operating from self-storage units that have policies prohibiting alcohol storage. This and the potential for increased alcohol accessibility online have implications for public health given the relationship between alcohol’s availability and consumption or harm. There is a need to ensure that regulations for delivery are adequate for protecting children and vulnerable adults. The Licensing Act 2003 may require modernization in the digital age. Future research must explore a relationship between online alcohol availability and deprivation.

## Introduction

Alongside the price and marketing of alcohol, alcohol consumption or harm is influenced by availability [1]. Online alcohol sales have increased and accelerated since the COVID-19 pandemic [2], and third-party platforms (hereinafter “platforms”), such as Deliveroo, that offer alcohol have expanded [3,4]. These platforms offer rapid delivery and mediate sales and delivery, meaning the license holder is not always the person delivering alcohol and undertaking age verification. Preventing the purchase of alcohol to children is important.

Increased availability of alcohol through platforms is relevant to public health because higher off-premise alcohol outlet density is associated with increased alcohol consumption or harm, which is stronger in more deprived areas [1,5]. Similar harms are associated with increasing opening hours [1]. Preliminary evidence shows that online platforms offer 24-hour services or late-night delivery [6] when physical shops might not be open. Selling alcohol online is not new, but late-night rapid delivery is. If the availability of alcohol on platforms increases the total availability and accessibility of alcohol, this might lead to increases in alcohol consumption or harm. Additional concerns arise regarding age verification undertaken by third-party delivery drivers [7].

This study aimed to describe alcohol outlet availability on the platform Deliveroo and explore whether availability was associated with deprivation. We also aimed to understand the proportion of outlets also selling tobacco or e-cigarettes, those that used Deliveroo-employed drivers, and compare listed addresses on Deliveroo with physical offline outlets.

## Methods

We conducted an exploratory, cross-sectional study across London, England, using data from Deliveroo.

### Data

We extracted the number of outlets offering alcohol on Deliveroo across 32 London boroughs (excluding City of London due to a small population). London was selected pragmatically. Deliveroo was chosen as it is one of the United Kingdom’s most popular platforms [8], and preliminary assessments showed that it had more alcohol outlets compared with similar platforms.

Eligible outlets delivered to a London borough and predominantly offered alcohol. We excluded restaurants offering preprepared food and supermarkets offering fruits or vegetables. Eligible outlets either exclusively offered alcohol or offered alcohol alongside confectionary or snacks (outlets most relevant to public health). We coded outlets as those that exclusively sold alcohol (eg, Alcohol4U), off-licenses, and premium (eg, bottle or wine shops).

### Procedure

We determined outlet eligibility and data extraction concurrently (July-August 2023) after pilot testing, which revealed high inter-rater agreement (88% for assigning eligibility and 98% for accuracy of extracted data).

Using Chrome, we used Deliveroo’s built-in alcohol filter to identify eligible outlets. One researcher checked eligibility and identified whether outlets sold tobacco or e-cigarettes and what type of driver was used. A randomly selected 10% of the data were checked by a second researcher, with high agreement (≥97% for assigning eligibility and data extraction checking). If we were uncertain about an outlet’s eligibility or extraction, we sought consensus.

For 60 outlets delivering to the most boroughs, we compared the address listed on Deliveroo with the address in Google to identify whether the Deliveroo outlet also had an offline outlet.

### Analysis

We converted the total number of outlets into a crude rate per 1000 population aged 18-64 years for all boroughs and outlet types using 2021 midyear population estimates [9]. Pearson correlation coefficients were calculated to explore the association between the crude rate of outlets (overall and by outlet type) and average Indices of Multiple Deprivation (IMD) 2019 scores [10]. Descriptive analyses were undertaken in Excel (Microsoft Corp), and maps were developed in RStudio (R-version 4.2.2; Posit PBC).

### Ethical Considerations

Ethical approval was not required as included data were publicly available, not individual level, and were deidentified.

## Results

**Figure 1:**
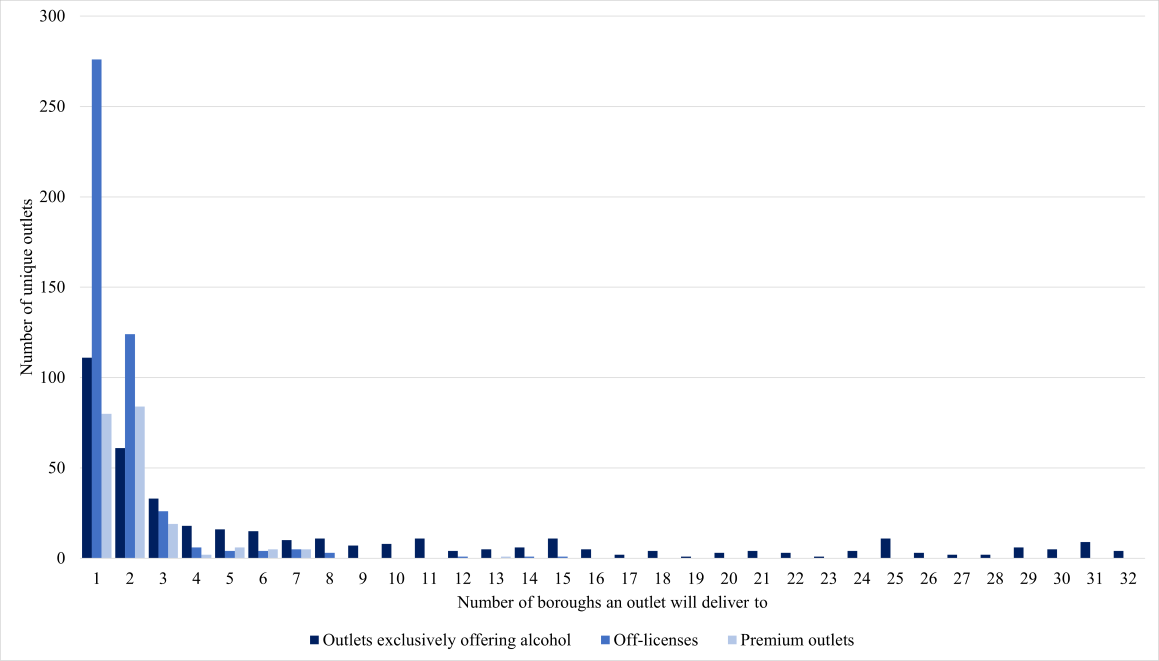
Distribution of the number of unique outlets offering alcohol on Deliveroo at the time of data collection (July-August 2023) by the number of London boroughs that an outlet reported delivering to and outlet type (N=1049).

Overall, 4277 outlets offered alcohol on Deliveroo across London: 72.2% (3086/4277) exclusively offered alcohol, 18.0% (770/4277) were off-licenses, and 9.8% (421/4277) were premium outlets (Table 1). When we removed duplicate outlets (outlets that delivered to more than 1 borough), a lower proportion of these 1049 unique outlets exclusively offered alcohol (n=396, 37.8%). This difference is because more outlets exclusively offering alcohol delivered across multiple boroughs (Figure 1).

**Table 1:** The number and percentage of total alcohol outlets listed on Deliveroo at the time of data collection (July-August 2023) by London borough and outlet type (N=4277)^a^.

Borough	Outlets exclusively offering alcohol, n (%)	Off-licenses, n (%)	Premium outlets, n (%)	Total, n (%)
Kensington and Chelsea	144 (62.6)	43 (18.7)	43 (18.7)	230 (5.4)
Hammersmith and Fulham	145 (71.1)	35 (17.2)	24 (11.8)	204 (4.8)
Westminster	135 (65.9)	34 (16.6)	36 (17.6)	205 (4.8)
Islington	135 (63.1)	45 (21.0)	34 (15.9)	214 (5.0)
Camden	132 (67.7)	37 (19.0)	26 (13.3)	195 (4.6)
Richmond upon Thames	118 (81.9)	19 (13.2)	7 (4.9)	144 (3.4)
Kingston upon Thames	105 (83.3)	17 (13.5)	4 (3.2)	126 (2.9)
Hackney	123 (66.5)	23 (12.4)	39 (21.1)	185 (4.3)
Merton	97 (74.0)	27 (20.6)	7 (5.3)	131 (3.1)
Southwark	126 (66.0)	38 (19.9)	27 (14.1)	191 (4.5)
Wandsworth	132 (66.0)	39 (19.5)	29 (14.5)	200 (4.7)
Lambeth	124 (63.6)	42 (21.5)	29 (14.9)	195 (4.6)
Haringey	96 (66.2)	32 (22.1)	17 (11.7)	145 (3.4)
Harrow	112 (87.5)	15 (11.7)	1 (0.8)	128 (3.0)
Tower Hamlets	120 (67.4)	30 (16.9)	28 (15.7)	178 (4.2)
Ealing	143 (79.4)	25 (13.9)	12 (6.7)	180 (4.2)
Sutton	80 (86.0)	12 (12.9)	1 (1.1)	93 (2.2)
Hounslow	105 (80.8)	21 (16.2)	4 (3.1)	130 (3.0)
Greenwich	99 (75.0)	21 (15.9)	12 (9.1)	132 (3.1)
Brent	121 (82.3)	22 (15.0)	4 (2.7)	147 (3.4)
Lewisham	86 (70.5)	22 (18.0)	14 (11.5)	122 (2.9)
Waltham Forest	91 (82.7)	12 (10.9)	7 (6.4)	110 (2.6)
Croydon	79 (67.5)	33 (28.2)	5 (4.3)	117 (2.7)
Newham	92 (86.0)	11 (10.3)	4 (3.7)	107 (2.5)
Barking and Dagenham	30 (53.6)	25 (44.6)	1 (1.8)	56 (1.3)
Hillingdon	61 (78.2)	17 (21.8)	0 (0.0)	78 (1.8)
Bromley	68 (85.0)	10 (12.5)	2 (2.5)	80 (1.9)
Redbridge	50 (73.5)	16 (23.5)	2 (2.9)	68 (1.6)
Bexley	30 (73.2)	9 (22.0)	2 (4.9)	41 (1.0)
Havering	23 (54.8)	19 (45.2)	0 (0.0)	42 (1.0)
Enfield	36 (73.5)	13 (26.5)	0 (0.0)	49 (1.1)
Barnet	48 (88.9)	6 (11.1)	0 (0.0)	54 (1.3)
Total	3086 (72.2)	770 (18.0)	421 (9.8)	4277 (100)

^a^The table is ordered from the highest crude rate of total alcohol outlets to the lowest. Outlets are counted in more than 1 borough if they deliver to multiple boroughs.

Most outlets exclusively offering alcohol used drivers not employed by Deliveroo (2887/3086, 93.6%; Table 2). The inverse was true for premium outlets (50/421, 11.9%) and off-licenses (73/770, 9.5%). Tobacco or e-cigarettes were offered alongside alcohol in 95.6% (2951/3086) of outlets exclusively offering alcohol (≥90% in 31/32 boroughs) and in 76.4% (588/770) of off-licenses (Table S1 in Multimedia Appendix 1).

The crude rate of web-based alcohol outlets per 1000 population aged 18-64 years was 0.73, ranging from 0.22 (Barnet) to 2.29 (Kensington and Chelsea); see Table 3 and Figures S1-S4 in Multimedia Appendix 1). The crude rate of outlets exclusively offering alcohol was higher than the rate of off-licenses or premium outlets across every borough. The crude rate of premium outlets was higher than the rate of off-license only in Hackney and Westminster.

Pearson correlations revealed no association between the average borough IMD score and the crude rate of total outlets (*r*(30)=–0.03, *P*=.87) nor for outlets exclusively offering alcohol (*r*(30)=–0.15, *P*=.41), off-licenses (*r*(30)=0.10, *P*=.58), or premium outlets (*r*(30)=0.24, *P*=.18).

We were able to locate a match for the listed addresses for 100% (20/20) of outlets exclusively offering alcohol, 95% (19/20) of off-licenses, and 85% (17/20) of premium outlets. Self-storage addresses were listed for 85% (17/20) of outlets exclusively offering alcohol compared with 11% (2/19) and 12% (2/17) of off-license and premium outlets, respectively.

**Table 2:** The number and percentage of total alcohol outlets listed on Deliveroo at the time of data collection (July-August 2023) using drivers not employed by Deliveroo, by London borough and outlet type (N=3010)^a^.

Borough	Outlets exclusively offering alcohol, n (%)	Off-licenses, n (%)	Premium outlets, n (%)	Total, n (%)
Kensington and Chelsea	136 (94.4)	3 (7.0)	3 (7.0)	142 (61.7)
Hammersmith and Fulham	136 (93.8)	4 (11.4)	2 (8.3)	142 (69.6)
Westminster	129 (95.6)	4 (11.8)	3 (8.3)	136 (66.3)
Islington	127 (94.1)	5 (11.1)	2 (5.9)	134 (62.6)
Camden	125 (94.7)	4 (10.8)	3 (11.5)	132 (67.7)
Richmond upon Thames	113 (95.8)	6 (31.6)	1 (14.3)	120 (83.3)
Kingston upon Thames	97 (92.4)	1 (5.9)	1 (25.0)	99 (78.6)
Hackney	112 (91.1)	4 (17.4)	2 (5.1)	118 (63.8)
Merton	90 (92.8)	0 (0.0)	1 (14.3)	91 (69.5)
Southwark	123 (97.6)	4 (10.5)	3 (11.1)	130 (68.1)
Wandsworth	126 (95.5)	7 (17.9)	1 (3.4)	134 (67.0)
Lambeth	121 (97.6)	4 (9.5)	3 (10.3)	128 (65.6)
Haringey	90 (93.8)	3 (9.4)	2 (11.8)	95 (65.5)
Harrow	101 (90.2)	0 (0.0)	1 (100.0)	102 (79.7)
Tower Hamlets	107 (89.2)	4 (13.3)	3 (10.7)	114 (64.0)
Ealing	130 (90.9)	4 (16.0)	2 (16.7)	136 (75.6)
Sutton	73 (91.3)	0 (0.0)	1 (100.0)	74 (79.6)
Hounslow	97 (92.4)	2 (9.5)	1 (25.0)	100 (76.9)
Greenwich	94 (94.9)	3 (14.3)	3 (25.0)	100 (75.8)
Brent	115 (95.0)	1 (4.5)	2 (50.0)	118 (80.3)
Lewisham	81 (94.2)	2 (9.1)	3 (21.4)	86 (70.5)
Waltham Forest	86 (94.5)	2 (16.7)	2 (28.6)	90 (81.8)
Croydon	72 (91.1)	1 (3.0)	2 (40.0)	75 (64.1)
Newham	87 (94.6)	1 (9.1)	2 (50.0)	90 (84.1)
Barking and Dagenham	28 (93.3)	2 (8.0)	0 (0.0)	30 (53.6)
Hillingdon	58 (95.1)	0 (0.0)	N/A^b^	58 (74.4)
Bromley	64 (94.1)	0 (0.0)	1 (50.0)	65 (81.3)
Redbridge	46 (92.0)	0 (0.0)	0 (0.0)	46 (67.6)
Bexley	26 (86.7)	0 (0.0)	0 (0.0)	26 (63.4)
Havering	18 (78.3)	2 (10.5)	N/A	20 (47.6)
Enfield	35 (97.2)	0 (0.0)	N/A	35 (71.4)
Barnet	44 (91.7)	0 (0.0)	N/A	44 (81.5)
Total	2887 (93.6)	73 (9.5)	50 (11.9)	3010 (70.4)

^a^The table is ordered from the highest crude rate of total alcohol outlets to the lowest. Outlets are counted in more than 1 borough if they deliver to multiple boroughs.

^b^N/A: not applicable (there were no outlets of this type for the borough).

**Table 3:** The crude rates of total alcohol outlets per 1000 population aged 18-64 years listed on Deliveroo at the time of data collection (July-August 2023) by London borough and outlet type^a^.

Borough	Exclusively offering alcohol	Off-licenses	Premium outlets	Total alcohol outlets
Kensington and Chelsea	1.44	0.43	0.43	2.29
Hammersmith and Fulham	1.10	0.26	0.18	1.54
Westminster	0.90	0.23	0.24	1.37
Islington	0.84	0.28	0.21	1.34
Camden	0.89	0.25	0.17	1.31
Richmond upon Thames	0.99	0.16	0.06	1.20
Kingston upon Thames	0.98	0.16	0.04	1.18
Hackney	0.67	0.13	0.21	1.01
Merton	0.69	0.19	0.05	0.93
Southwark	0.56	0.17	0.12	0.86
Wandsworth	0.56	0.16	0.12	0.84
Lambeth	0.53	0.18	0.12	0.83
Haringey	0.53	0.18	0.09	0.80
Harrow	0.69	0.09	0.01	0.79
Tower Hamlets	0.52	0.13	0.12	0.77
Ealing	0.59	0.10	0.05	0.75
Sutton	0.62	0.09	0.01	0.72
Hounslow	0.56	0.11	0.02	0.69
Greenwich	0.51	0.11	0.06	0.68
Brent	0.54	0.10	0.02	0.65
Lewisham	0.42	0.11	0.07	0.59
Waltham Forest	0.49	0.06	0.04	0.59
Croydon	0.32	0.13	0.02	0.47
Newham	0.38	0.05	0.02	0.44
Barking and Dagenham	0.22	0.18	0.01	0.41
Hillingdon	0.32	0.09	0.00	0.41
Bromley	0.34	0.05	0.01	0.40
Redbridge	0.26	0.08	0.01	0.35
Bexley	0.20	0.06	0.01	0.28
Havering	0.15	0.12	0.00	0.27
Enfield	0.18	0.06	0.00	0.24
Barnet	0.20	0.02	0.00	0.22
Total	0.53	0.13	0.07	0.73

^a^The table is ordered from the highest crude rate of total alcohol outlets to the lowest. Outlets are counted in more than 1 borough if they deliver to multiple boroughs.

## Discussion

### Principal Findings

A large number of web-based alcohol outlets deliver alcohol in London, particularly from outlets exclusively offering alcohol alongside tobacco or e-cigarettes. Most outlets exclusively offering alcohol delivered across multiple boroughs and used drivers not employed by Deliveroo. Among the small number of outlets checked, many listed their premise as a self-storage address. We did not collect data on operating hours, but a small-scale London-based study found that similar online outlets, particularly those almost exclusively offering alcohol and tobacco or e-cigarettes, tend to be open late into the evening or early morning [6], beyond hours of brick-and-mortar outlets. These findings are concerning given that higher availability of alcohol is associated with higher alcohol consumption or harm [1,5].

To sell alcohol in England, outlets must be licensed under the Licensing Act 2003 (herein the Act), and license holders have a legal responsibility to refuse sale to children or intoxicated people [11]. The Act was written when alcohol sales overwhelmingly occurred in person. Although Deliveroo’s policy requires drivers to age verify and refuse delivery to an intoxicated person [12], they hold no legal responsibility under the Act (this remains with the license holder) [11]. This might lead to poorer age verification. Evidence suggests that delivery processes of online sales (such as age verification) do not have the same standards as physical outlets [7]. Additionally, 45% of mystery shoppers under-25 ordering age-restricted products using third-party online platforms were asked for proof of age on delivery compared with 78% in person [13]. If the license holder employs their own drivers to undertake deliveries, age verification might be more similar to in person because the license holder (or employee) is interacting with the customer.

Among the small number of outlets checked, many listed self-storage addresses, despite these companies prohibiting alcohol storage (e.g. [14]). Although the Act does not prohibit such locations as licensable premises, regulators may be unaware and should consider whether this is appropriate and what safeguards are required to protect children and vulnerable people. Platforms do not only increase convenience of existing alcohol retailers but also facilitate the proliferation of new, online-only outlets, thereby increasing overall alcohol availability.

Previous UK research reports significant associations between availability of physical alcohol outlets and deprivation [15,16]. Existing research, mostly on food, reports higher availability of online outlets with higher deprivation [17]. Availability and deprivation were not associated in our study. Deprivation varies markedly within London boroughs, so our finding may reflect the use of borough, not postcode, level deprivation measures. A similar New Zealand study reported no significant relationship between online alcohol access and deprivation [18]. Outlets exclusively offering alcohol tended to deliver across multiple boroughs, some from central warehouses serving most of London. There might be sociodemographic differences in people who use online platforms for alcohol compared with those who buy in person; however, little research exists and further exploration is required. Similarly, more research comprehensively describing the online alcohol retail environment would aid the call of Directors of Public Health to use licensing to prevent alcohol-related harm [19].

### Limitations

This study reports the presence of alcohol and tobacco or e-cigarettes offered via the web and not differences in the number of products available at each outlet. We do not capture actual sales of products. We underrepresent the availability of alcohol on Deliveroo by excluding supermarkets and restaurants and did not capture other relevant aspects of availability related to alcohol consumption and harm such as pricing or operating hours. Our results are from 1 metropolitan English region, so they may not be generalizable to other regions or countries.

### Conclusions

The digital age revolutionized the home delivery industry. Because increased availability of alcohol is associated with increased alcohol consumption or alcohol-related harm, understanding alcohol’s availability online is a public health priority. We found many outlets offering rapid-delivery alcohol, often delivered by drivers who have no legal responsibility for age verification. Regulations for delivery are needed to protect children and vulnerable adults. We found online outlets listing self-storage facilities, testing the boundaries of the licensing system. More research in this area is justified, and the Licensing Act 2003 requires modernization in the digital age.

## Appendix

Multimedia Appendix 1 The percentage and number of total alcohol outlets and crude rate of outlets.

## Abbreviations

IMD: Indices of Multiple Deprivation

## Data Availability

The data sets generated during and analyzed during this study are available from the corresponding author on reasonable request.
