# Supplementary material for: Availability of Alcohol on an Online Third-Party Delivery Platform Across London Boroughs, England: Exploratory Cross-Sectional Study
Source: JMIR Form Res. 2024 Jun 28;8:e54587. doi: 10.2196/54587 (PMC11245658; doi:10.2196/54587)

Multimedia Appendix 1: The percentage and number of total alcohol outlets and crude rate of outlets

Table S1. The percentage and number of total alcohol outlets listed on Deliveroo at the time of data collection (July-August 2023) that also offer tobacco/e-cigarettes by London borough and outlet type (n=3,576)

| *London Borough* | *% of outlets exclusively offering alcohol (n)* | *% of off-licenses (n)* | *% of premium outlets (n)* | *% of total alcohol outlets (n)* |
| --- | --- | --- | --- | --- |
| Kensington and Chelsea | 97.2 (140) | 81.4 (35) | 7.0 (3) | 77.4 (178) |
| Hammersmith and Fulham | 94.5 (137) | 82.9 (29) | 8.3 (2) | 82.4 (168) |
| Westminster | 97.8 (132) | 82.4 (28) | 5.6 (2) | 79.0 (162) |
| Islington | 94.8 (128) | 80.0 (36) | 5.9 (2) | 77.6 (166) |
| Camden | 96.2 (127) | 81.1 (30) | 7.7 (2) | 81.5 (159) |
| Richmond upon Thames | 95.8 (113) | 89.5 (17) | 28.6 (2) | 91.7 (132) |
| Kingston upon Thames | 95.2 (100) | 82.4 (14) | 25.0 (1) | 91.3 (115) |
| Hackney | 92.7 (114) | 65.2 (15) | 2.6 (1) | 70.3 (130) |
| Merton | 93.8 (91) | 77.8 (21) | 14.3 (1) | 86.3 (113) |
| Southwark | 96.8 (122) | 71.1 (27) | 7.4 (2) | 79.1 (151) |
| Wandsworth | 96.2 (127) | 79.5 (31) | 10.3 (3) | 80.5 (161) |
| Lambeth | 96.8 (120) | 73.8 (31) | 6.9 (2) | 78.5 (153) |
| Haringey | 95.8 (92) | 71.9 (23) | 5.9 (1) | 80.0 (116) |
| Harrow | 96.4 (108) | 86.7 (13) | 100.0 (1) | 95.3 (122) |
| Tower Hamlets | 92.5 (111) | 60.0 (18) | 3.6 (1) | 73.0 (130) |
| Ealing | 94.4 (135) | 84.0 (21) | 8.3 (1) | 87.2 (157) |
| Sutton | 95.0 (76) | 91.7 (11) | 100.0 (1) | 94.6 (88) |
| Hounslow | 95.2 (100) | 61.9 (13) | 50.0 (2) | 88.5 (115) |
| Greenwich | 97.0 (96) | 61.9 (13) | 8.3 (1) | 83.3 (110) |
| Brent | 96.7 (117) | 90.9 (20) | 25.0 (1) | 93.9 (138) |
| Lewisham | 97.7 (84) | 77.3 (17) | 7.1 (1) | 83.6 (102) |
| Waltham Forest | 96.7 (88) | 50.0 (6) | 14.3 (1) | 86.4 (95) |
| Croydon | 94.9 (75) | 81.8 (27) | 20.0 (1) | 88.0 (103) |
| Newham | 96.7 (89) | 63.6 (7) | 25.0 (1) | 90.7 (97) |
| Barking and Dagenham | 93.3 (28) | 80.0 (20) | 0.0 (0) | 85.7 (48) |
| Hillingdon | 96.7 (59) | 64.7 (11) | NA | 89.7 (70) |
| Bromley | 94.1 (64) | 70.0 (7) | 50.0 (1) | 90.0 (72) |
| Redbridge | 94.0 (47) | 56.3 (9) | 0.0 (0) | 82.4 (56) |
| Bexley | 96.7 (29) | 100.0 (9) | 0.0 (0) | 92.7 (38) |
| Havering | 87.0 (20) | 63.2 (12) | NA | 76.2 (32) |
| Enfield | 100.0 (36) | 92.3 (12) | NA | 98.0 (48) |
| Barnet | 95.8 (46) | 83.3 (5) | NA | 94.4 (51) |
| *Total* | *95.6 (2,951)* | *76.4 (588)* | *8.8 (37)* | *83.6 (3,576)* |

n: number; NA: not applicable (there were no outlet of this type for the borough)

Figure S1. Crude rate of total outlets offering alcohol on Deliveroo at the time of data collection (July-August 2023) per 1,000 population aged 18-64 years, by London borough


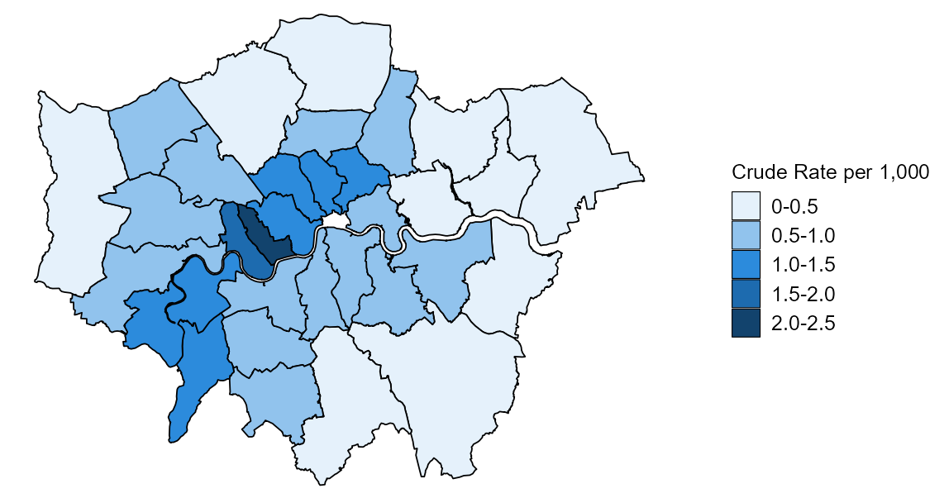


Figure S2. Crude rate of outlets exclusively offering alcohol on Deliveroo at the time of data collection (July-August 2023) per 1,000 population aged 18-64 years, by London borough


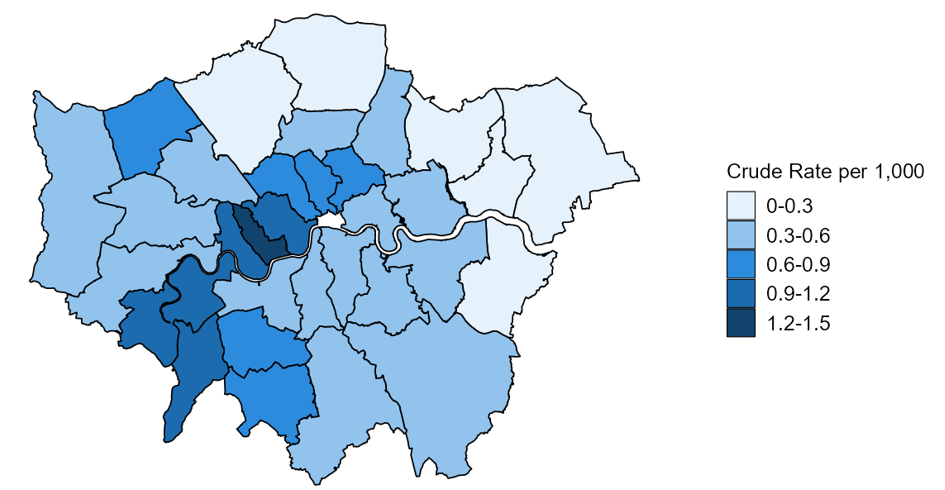


Figure S3. Crude rate of off-licenses offering alcohol on Deliveroo at the time of data collection (July-August 2023) per 1,000 population aged 18-64 years, by London borough


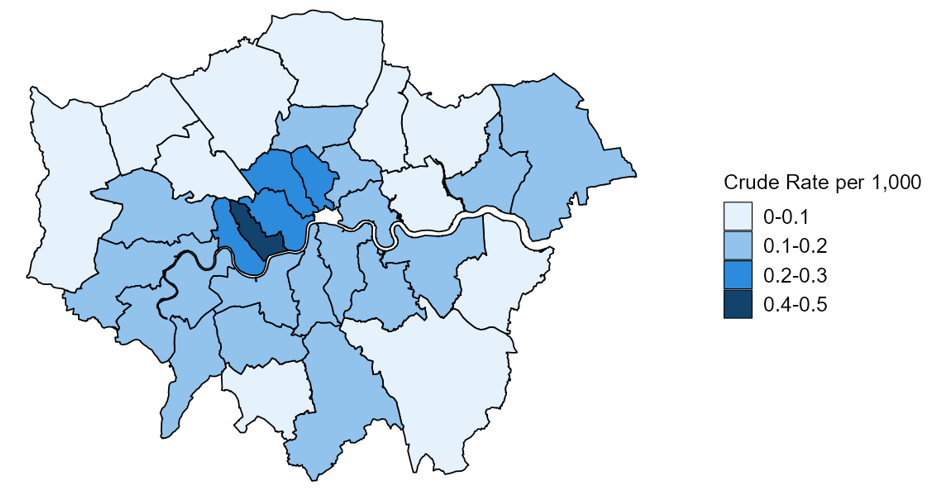


Figure S4. Crude rate of premium outlets offering alcohol on Deliveroo at the time of data collection (July-August 2023) per 1,000 population aged 18-64 years, by London borough


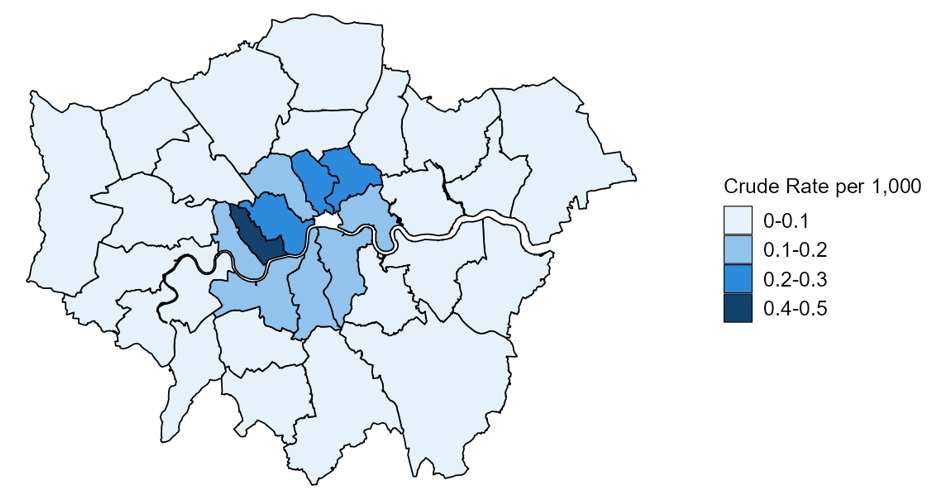

Supplement: Multimedia Appendix 1 [file formative_v8i1e54587_app1.docx]
